# Oxygenation management during veno-arterial ECMO support for cardiogenic shock: a multicentric retrospective cohort study

**DOI:** 10.1186/s13613-024-01286-2

**Published:** 2024-04-10

**Authors:** Hadrien Winiszewski, Thibault Vieille, Pierre-Grégoire Guinot, Nicolas Nesseler, Mael Le Berre, Laure Crognier, Anne-Claude Roche, Jean-Luc Fellahi, Nicolas D’Ostrevy, Zied Ltaief, Juliette Didier, Osama Abou Arab, Simon Meslin, Vincent Scherrer, Guillaume Besch, Alexandra Monnier, Gael Piton, Antoine Kimmoun, Gilles Capellier

**Affiliations:** 1 Service de réanimation médicale, CHU Besançon, Besançon, France; 2 Service d’anesthésie-réanimation chirurgicale, CHU Dijon, Dijon, France; 3 Department of Anesthesia and Critical Care, University Hospital of Rennes, Pontchaillou, Rennes, France; 4 Intensive Care Unit, Anesthesia and Critical Care Department, Rangueil University Hospital, Toulouse, France; 5 Anesthesia, Intensive Care and Perioperative Medicine, Nouvel Hôpital Civil, Strasbourg University Hospital, Strasbourg, France; 6 Service d’Anesthésie-Réanimation, Hôpital Louis Pradel, Hospices Civils de Lyon, Lyon, France; 7 Cardiac Surgery Department, Montpied Hospital, University Hospital of Clermont-Ferrand, Clermont-Ferrand, France; 8 Department of Adult Intensive Care Medicine, Lausanne University Hospital and Lausanne University, Lausanne, 1011 Switzerland; 9 Service de médecine intensive réanimation, CHU Pitié Salpêtrière, Paris, France; 10 Department of Anaesthesia and Critical Care Medicine, Amiens University Medical Center, Amiens, France; 11 Anesthesiology and Critical Care Medicine Department, Hôpital Européen Georges Pompidou, APHP, Paris, France; 12 Department of Anaesthesiology and Critical Care, CHU Rouen, Rouen, F-76000 France; 13 Département d’Anesthésie Réanimation Chirurgicale, Université de Franche-Comté, CHU Besançon, CIC Inserm 1431, Besançon, EA3920, F-25000 France; 14 Service de Médecine Intensive-Réanimation Médicale, CHU Strasbourg, Nouvel Hôpital Civil, Université de Strasbourg, Strasbourg, 67000 France; 15 Service de médecine intensive réanimation, CHU Nancy, Créteil, France; 16 Department of Epidemiology and Preventive Medicine, School of Public Health and Preventive Medicine, Faculty of Medicine, Nursing and Health Sciences, Clayton, Australia; 17 Research Unit EA 3920 and SFR FED 4234, University of Franche Comté, Besancon, France

**Keywords:** Extracorporeal membrane oxygenation, Cardiogenic shock, Hyperoxia, Sweep gas oxygen fraction, Mortality

## Abstract

**Backgound:**

Hyperoxemia is common and associated with poor outcome during veno-arterial extracorporeal membrane oxygenation (VA ECMO) support for cardiogenic shock. However, little is known about practical daily management of oxygenation. Then, we aim to describe sweep gas oxygen fraction (F_S_O_2_), postoxygenator oxygen partial pressure (P_POST_O_2_), inspired oxygen fraction (F_I_O_2_), and right radial arterial oxygen partial pressure (P_a_O_2_) between day 1 and day 7 of peripheral VA ECMO support. We also aim to evaluate the association between oxygenation parameters and outcome. In this retrospective multicentric study, each participating center had to report data on the last 10 eligible patients for whom the ICU stay was terminated. Patients with extracorporeal cardiopulmonary resuscitation were excluded. Primary endpoint was individual mean F_S_O_2_ during the seven first days of ECMO support (F_S_O_2_ _*mean (day 1−7)*_).

**Results:**

Between August 2019 and March 2022, 139 patients were enrolled in 14 ECMO centers in France, and one in Switzerland. Among them, the median value for F_S_O_2_ _*mean (day 1−7)*_ was 70 [57; 79] % but varied according to center case volume. Compared to high volume centers, centers with less than 30 VA-ECMO runs per year were more likely to maintain F_S_O_2_ ≥ 70% (OR 5.04, CI 95% [1.39; 20.4], *p* = 0.017). Median value for right radial P_a_O_2_ _*mean (day 1−7)*_ was 114 [92; 145] mmHg, and decreased from 125 [86; 207] mmHg at day 1, to 97 [81; 133] mmHg at day 3 (*p* < 0.01). Severe hyperoxemia (i.e. right radial P_a_O_2_ ≥ 300 mmHg) occurred in 16 patients (12%). P_POST_O_2_, a surrogate of the lower body oxygenation, was measured in only 39 patients (28%) among four centers. The median value of P_POST_O_2_ _*mean (day 1−7)*_ value was 198 [169; 231] mmHg. By multivariate analysis, age (OR 1.07, CI95% [1.03–1.11], *p* < 0.001), F_S_O_2_ _*mean (day 1−3)*_(OR 1.03 [1.00-1.06], *p* = 0.039), and right radial P_a_O_2_ _*mean (day 1−3)*_ (OR 1.03, CI95% [1.00-1.02], *p* = 0.023) were associated with in-ICU mortality.

**Conclusion:**

In a multicentric cohort of cardiogenic shock supported by VA ECMO, the median value for F_S_O_2_ _*mean (day 1−7)*_ was 70 [57; 79] %. P_POST_O_2_ monitoring was infrequent and revealed significant hyperoxemia. Higher F_S_O_2_ _*mean (day 1−3)*_ and right radial P_a_O_2_ _*mean (day 1−3)*_ were independently associated with in-ICU mortality.

**Supplementary Information:**

The online version contains supplementary material available at 10.1186/s13613-024-01286-2.

## Background

While Veno-Arterial Extracorporeal Membrane Oxygenation (VA-ECMO) is primarily used to restore adequate tissue perfusion by increasing systemic blood flow, it also significantly impacts blood oxygenation because of the oxygenator integrated into the circuit. Indeed, severe hyperoxemia (i.e. P_a_O_2_ ≥ 300 mmHg) is commonly reported during VA-ECMO support, with prevalence ranging from 12 to 89% during the first 24 h [1–9]. Several studies have reported an association between severe hyperoxemia and poor outcome in this population, especially after refractory cardiac arrest [2–7, 9–11]. In the setting of cardiogenic shock rescued by VA-ECMO, although initial studies did not find such association [1, 7], there is emerging data supporting the link between hyperoxemia and mortality [8, 12, 13].

Based on these observational studies, the 2021 ELSO guidelines have recommended to target slight hyperoxemia after the oxygenator (P_POST_O_2_ around 150 mmHg) and to avoid hypoxemia on the right radial artery [14]. Such guidelines open to wide variation in clinical practice, as it is still unknown how to reach these oxygenation’s targets. Indeed, because of the dual circulation, right radial P_a_O_2_ is impacted by both the ventilator settings (inspired fraction of oxygen (F_I_O_2_) and positive end expiratory pressure (PEEP)), the ECMO blood flow, and the sweep gas oxygen fraction (F_S_O_2_) [15, 16].

To date, there is very limited data on oxygenation management during VA-ECMO support for cardiogenic shock, all studies reporting monocentric or bicentric experiences, and limited to the first 24 h [7, 8], 48 h [12], or 72 h [1]. Then, we aimed to describe the current oxygenation management for the first week of VA ECMO support in a multicentric cohort of patients with cardiogenic shock. We also aimed to evaluate the association between oxygenation parameters and outcome.

## Methods

This was a retrospective cohort multicentric study conducted in 14 intensive care units (ICU) in France, and one ICU in Switzerland. The study was approved by our institutional review board in august 2021 (“*ECMOxy: oxygenation practice in patients with cardiogenic shock supported by VA ECMO*”, approval number EI/2021/1061). This was considered as a multicentric Evaluation of Professional Practices. Aiming at improving quality of care, this French legal framework allows collection of anonymized data related to standard care without need of written patient’s consent. However, in Switzerland, the consent of the patient or his surrogate was mandatory. The research was performed in accordance with the ethical standards in the 1964 Declaration of Helsinki and its later amendments.

### Patients

Inclusion criteria were adult patients, supported by VA-ECMO for refractory cardiogenic shock, and having available data on F_S_O_2_ from the day of implantation (day 1) to day 7 (or the day of weaning if before day 7). Exclusion criteria were extracorporeal cardiopulmonary resuscitation (ECPR), and ECMO duration less than 24 h. Each participating center had to report data on the last 10 eligible patients for whom the ICU stay was terminated.

### Data collection

The following data were collected: demographic data, characteristic of centers, indication for VA-ECMO support, VA-ECMO configuration, Simplified Acute Physiology Score 2 (SAPS2) and Sequential Organ Failure Assessment (SOFA) score, data related to extracorporeal oxygenation (F_S_O_2_ two times daily, and postoxygenator oxygen partial pressure (P_POST_O_2_) if available), data related to systemic oxygenation (F_I_O_2_, oxygen partial pressure on the right radial artery (P_a_O_2_), tidal volume, PEEP, and extubation), and data related to clinical outcome (need for renal replacement therapy, ECMO duration, ECMO weaning, LVAD implantation, heart transplantation, and in-ICU mortality, i.e. death during the same ICU stay than ECMO canulation). Oxygenation related data were reported from day 1 to day 7 of VA-ECMO support (or the day of weaning if before day 7).

### Endpoints

Primary endpoint was individual mean F_S_O_2_ during the seven first days of ECMO support (F_S_O_2 *mean* (*day 1−7)*_). Secondary endpoints were individual mean F_S_O_2_ during the three first days (F_S_O_2 __*mean* (*day 1−3)*_), individual mean right radial P_a_O_2_ during the seven first days (right radial P_a_O_2_ _*mean (day 1−7)*_), prevalence of P_POST_O_2_ monitoring, prevalence of extubation, and factors associated with in-ICU mortality.

### Statistical analysis

According to a Shapiro test, the studied variables were not normally distributed. Quantitative parameters were then described as median [Interquartile range] and number (percentage). F_S_O_2_ was dichotomized at the median value in the overall population (70%).

First, univariate analysis was performed to identify factors significantly associated with F_S_O_2 *mean (day 1-7)*_ ≥ 70% and with in-ICU mortality. Wilcoxon test was used to compare quantitative parameters and Chi-square test or Fisher exact test for qualitative parameters. A Wilcoxon signed rank test was used to compare repeated variables.

Second, we performed a multivariate logistic regression analysis to identify factors independently associated with F_S_O_2 *mean (day 1-7)*_ ≥ 70%. Variables associated with F_S_O_2 *mean (day 1-7)*_ ≥ 70% with a *p* value of less than 0.1 by univariate analysis were introduced in the model.

Third, we performed a multivariate logistic regression analysis to identify factors associated with in-ICU mortality. We entered factors associated with in-ICU mortality identified by univariate analysis as well as the duration of ECMO given its potential relevance. We then proceeded to a stepwise AIC backward regression. Multicollinearity between variables of the model was assessed using variance inflation factors. We evaluated the goodness of fit of logistic regression models with a Hosmer Lemeshow test. In case of missing data, no imputation was carried out because they were below 5%. A *p* value of less than 0.05 was considered statistically significant. Statistical analysis was performed with R version 4.0.3.

## Results

### Patients and centers

The first patient received VA ECMO in August 2019 and the last in March 2022. Although it was asked to report data for 10 patients, three centers reported data for 5 (5 consent withdrawals in a center in Switzerland), 9 (1 duplicate), and 11 patients, respectively. Data were available for 145 patients. Because oxygenation physiology is very different in peripheral and central ECMO, we secondarily decided to exclude the 6 patients with central ECMO, and 139 patients were finally analyzed.

Acute coronary syndrome was the main cause of cardiogenic shock (*n* = 50, 36%), followed by acutely decompensated cardiomyopathy (*n* = 44, 32%), and postcardiotomy shock (*n* = 26, 19%). ECMO was mainly inserted through femoral vessels (*n* = 129, 93%). ECMO was successfully weaned in 100 patients (72%), and 87 patients were discharged alive from ICU (63%). Baseline patient’s characteristics are reported in Table 1. Among the 15 participating centers, 4 used to manage less than 30 VA-ECMO patients/year, 9 between 30 and 100 VA ECMO patients/year, and 2 used to manage more than 100 VA ECMO patients/year.

**Table 1:** Baseline characteristics

Patients characteristics	Patients (*n* = 139)
Age (years)	57 [47;62]
SAPS2 score	60 [47;77]
SOFA score	10 [7;13]
*Number of patients according to centers case-volume per year*	
<30	32 (23%)
30–100	88 (63%)
>100	19 (14%)
*Indication for VA ECMO*	
Acute coronary syndrome	50 (36%)
Cardiomyopathy	44 (32%)
Postcardiotomy	26 (19%)
Pulmonary embolism	7 (5%)
Drug poisoning	7 (5%)
Others	5 (3%)
*ECMO configuration*	
Femoro-femoral	129 (93%)
Femoro-axillar	10 (7)
*Outcome*	
RRT during ICU stay	55 (40%)
ECMO duration (days)	6 [4–8]
ECMO weaning	100 (72%)
Bridge to LVAD	10 (7%)
Bridge to transplant	16 (12%)
ICU survival	87 (63%)

Data are number (percentage) and median [Interquartile range]. SAPS2: Simplified acute physiology score; SOFA: sequential organ failure assessment; LVAD: left ventricle assist device; RRT: renal replacement therapy

### Oxygenation management

#### F_S_O_2_

Among the 139 patients, the median value for F_S_O_2_ _*mean (day 1−7)*_ was 70 [57; 79] % and F_S_O_2_ did not differ between day 1 and day 3 (Wilcoxon signed rank test, *p* = 0.37). However, F_S_O_2_ _*mean (day 1−7)*_ varied between centers, ranging from 46 [43; 58] % to 84 [80; 92] %. Regarding to center case-volume, F_S_O_2_ _*mean (day 1−7)*_ was 72 [70; 81] %, 69 [57; 78] %, and 55 [44; 69] % in centers managing < 30, between 30 and 100, and > 100 VA ECMO per year, respectively (*p* < 0.01). F_S_O_2_ _*mean (day 1−7)*_ was 66 [50; 76] % in patients extubated during ECMO support, and 70 [59; 80] % in non-extubated patients (*p* = 0.04). In the whole cohort, 24 patients (17%) experienced at least one day with a F_S_O_2_ set at 100%. Descriptive data on oxygenation parameters are summarized in Table 2.

**Table 2:** Description of oxygenation parameters among the 139 patients supported by peripheral VA ECMO

Oxygenation parameters	F_S_O_2_ (%)		F_I_O_2_ (%)		Right radial P_a_O_2_ (mmHg)	
*Mean value*						
From day 1 to day 7 From day 1 to day 3	70 [57;79] 70 [60;80]		44 [35;57] 45 [35;60]		114 [92;145] 117 [90;158]	
*Daily evolution*						
Day 1 (*n = 139*) Day 2 (*n = 136*) Day 3 (*n = 132*) Day 4 (*n = 117*) Day 5 (*n = 97*) Day 6 (*n = 78*) Day 7 (*n = 68*)	70 [60;80]* 70 [60;80] 70 [57;80]* 70 [60;80] 70 [60;80] 70 [60;80] 70 [59;80]		50 [37;70]** 40 [30;60] 40 [30;60]** 40 [30;60] 40 [33;53] 40 [30;50] 40 [30;50]		125 [86;207]*** 106 [78;139] 97 [81;133]*** 94 [79;116] 96 [79;129] 88 [78;114] 95 [80;136]	
*Center case volume (per year)*	F_S_O_2__* mean (day 1−7)*_		F_I_O_2__* mean (day 1−7)*_		Right radial P_a_O_2__* mean (day 1−7)*_	
< 30 30–100 > 100	72 [70;81] 69 [57;78] 55 [44;69]	*p* < 0.01	46 [38;57] 47 [35;59] 36 [30;46]	*p* = 0.18	115 [92;155] 116 [96;147] 98 [87;112]	*p* = 0.04
*Extubation during ECMO support*	F_S_O_2__* mean (day 1−7)*_		F_I_O_2__* mean (day 1−7)*_		Right radial P_a_O_2__* mean (day 1−7)*_	
Yes No	66 [50;76] 70 [59;80]	*p* = 0.04	34 [29;39] 51 [42;64]	*p* < 0.01	102 [87;122] 118 [97;151]	*p* = 0.01

Data are median [Interquartile range]; F_S_O_2_: sweep gas oxygen fraction; F_I_O_2_: inspired oxygen fraction; p: p value

*Comparison between F_S_O_2 day 1_ and F_S_O_2 day 3_: *p* = 0.37

** Comparison between F_I_O_2 day 1_ and F_I_O_2 day 3_: *p* < 0.01

*** Comparison between right radial P_a_O_2 day 1_ and right radial P_a_O_2 day 3_: *p* < 0.01

By univariate analysis, center case-volume (*p* = 0.01), and right radial P_a_O_2_ _*mean (day 1−7)*_ (*p* < 0.01) were associated with F_S_O_2_ _*mean (day 1−7)*_ ≥ 70%. By multivariate analysis, centers with case-volume < 30 per year (OR 5.04, CI 95% [1.39; 20.4], *p* = 0.017), and right radial P_a_O_2_ _*mean (day 1−7)*_ (OR 1.01, CI 95% [1.00; 1.02], *p* = 0.006) were associated with F_S_O_2_ _*mean (day 1−7)*_ ≥ 70% (Supplementary Table 1).

#### F_I_O_2_

The median value of F_I_O_2_ _*mean (day 1−7)*_ was 44 [35; 57] %. There was no statistically difference in F_I_O_2_ according to center case volume (*p* = 0.18). Median value of F_I_O_2_ _*mean (day 1−7)*_ was 34 [29; 39] % in patients extubated at least one day during ECMO support, and 51 [42; 64] % in non-extubated patients (*p* < 0.01) (Table 2).

### Right radial P_a_O_2_

Among the 139 patients, 723 right radial P_a_O_2_ values were available during the seven first days of ECMO support. Data were missing for 7 patients, in whom P_a_O_2_ was monitored at the left radial artery.

Median value of right radial P_a_O_2 *mean (day 1-7)*_ was 114 [92; 145] mmHg. Right radial P_a_O_2_ decreased from 125 [86;207] mmHg at day 1, to 97 [81;133] mmHg at day 3 (Wilcoxon signed rank test *p* < 0.01). Regarding to center case volume right radial P_a_O_2 *mean (day 1-7)*_ was 115 [92;155], 116 [96;147] and 98 [87;112] mmHg in centers managing < 30, between 30 and 100, and > 100 VA ECMO per year, respectively (*p* = 0.04). Right radial P_a_O_2 *mean (day 1-7)*_ was 102 [87; 122] mmHg in patients extubated during ECMO support, and 118 [97; 151] mmHg in non-extubated patients (*p* = 0.01) (Table 2).

**Fig. 1:**
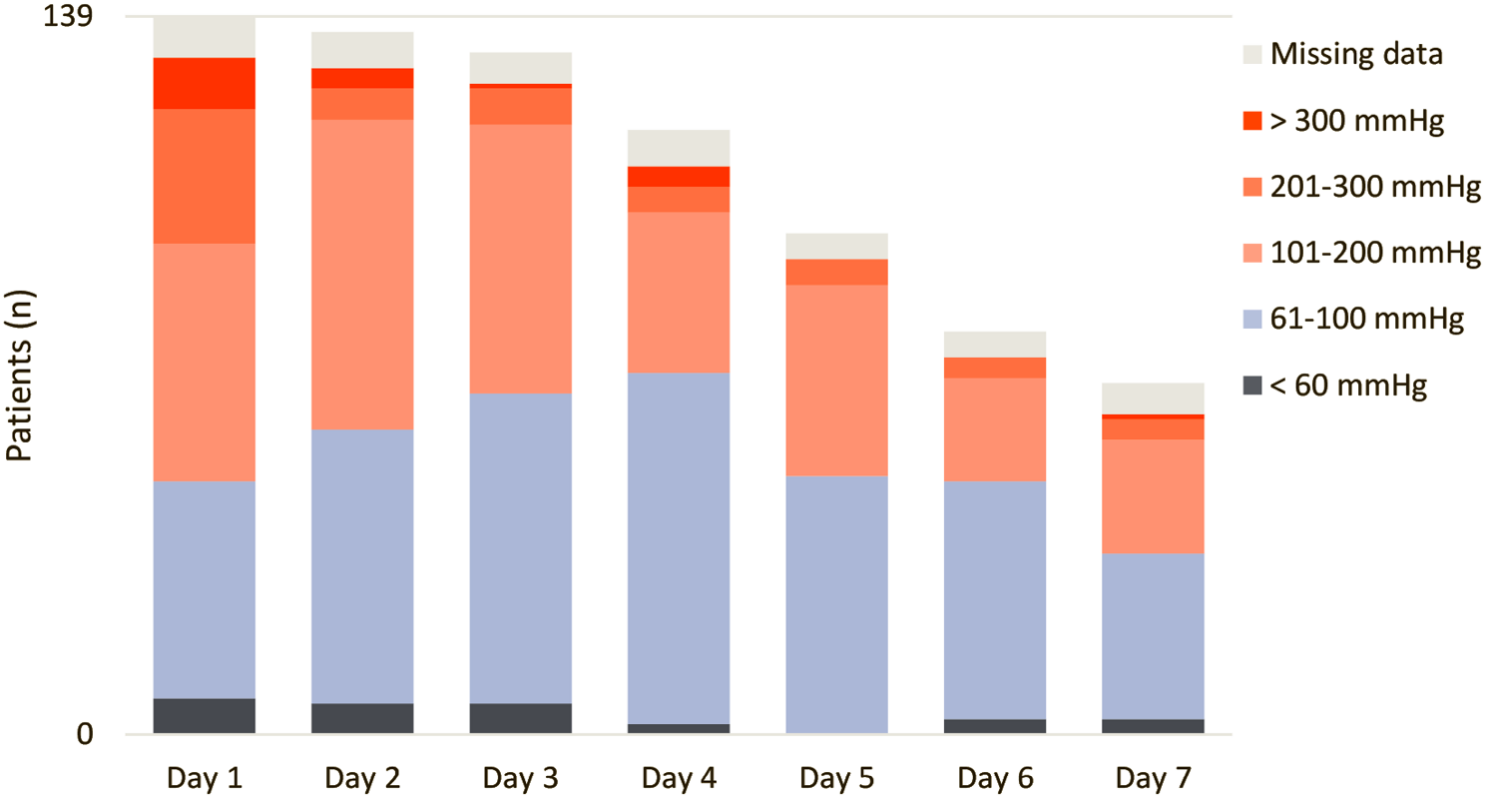
Right radial P_a_O_2_ ranges distribution

Among the 723 available right radial P_a_O_2_ values, 77 (11%) and 22 (3%) were ≥ 200 mmHg and ≥ 300 mmHg, respectively. Among the 139 patients, 16 (12%) experienced severe hyperoxemia, defined as at least one episode of right radial P_a_O_2_ ≥ 300 mmHg. Daily evolution of right radial P_a_O_2_ ranges distribution is reported in Fig. 1. Evolution of right radial P_a_O_2_ according to center case volume and outcome is presented in Supplementary Fig. 2.

### Tidal volume, PEEP, and extubation

On the day of ECMO implantation, 115 patients (83%) were intubated. Among them, median tidal volume and PEEP during the study period were 6.2 [5.7; 6.9] mL/kg of predicted body weight, and 7 [6; 9] cmH_2_O, respectively. During the seven first days of ECMO support, 47 patients (34%) were extubated at least one day. The median delay between ECMO start and extubation was 1 [0; 2] days.

### P_POST_O_2_

Among the 15 participating ICUs, 8 (53%) did not use to monitor daily P_POST_O_2_ and had no available data. In the remaining seven, 3 used to measure P_POST_O_2_ once daily but with F_S_O_2_ transiently increased at 100%. This aims at testing the membrane gas transfer capacity, rather than detecting ECMO-induced hyperoxemia of the lower part of the body. In the remaining four centers measuring P_POST_O_2_ maintaining F_S_O_2_ at its actual value, 39 patients (28%) had available data, and median value of P_POST_O_2_ _*mean (day 1−7)*_ value was 198 [169; 231] mmHg. Evolution of P_POST_O_2_ between day 1 and 7 is reported in Supplementary Fig. 1. Out of the 215 available P_POST_O_2_ values, 142 (66%) were above 150 mmHg. Among the 139 patients of the cohort, 44 (32%) had at least one day with F_S_O_2_ ≤ 50% without P_POST_O_2_ monitoring.

### Association of oxygenation parameters and in-ICU mortality

By univariate analysis, age (*p* < 0.01), SAPS2 score (*p* < 0.01), F_S_O_2 *day 2*_ (*p* = 0.02), F_S_O_2 *day 3*_ (*p* < 0.01), F_S_O_2_ _*mean (day 1−3)*_ (*p* < 0.01), F_S_O_2_ > 70% _*day 2*_ (i.e. at least one F_S_O_2_ value > 70% on day 2) (*p* = 0.03), F_S_O_2_ > 70% _*day 3*_ (*p* = 0.02), F_S_O_2_ > 70% _*day 1−3*_ (*p* = 0.04) and right radial P_a_O_2_ _*mean (day 1−3)*_ (*p* = 0.01) were associated with in-ICU mortality. We entered 5 variables in the multivariate logistic regression analysis: age, SAPS2, F_S_O_2_ _*mean (day 1−3)*_, and right radial P_a_O_2_ _*mean (day 1−3)*_; the duration of ECMO was also introduced in the model given its clinical relevance. Using a stepwise AIC backward regression analysis, three out of these five variables were conserved in the final model: age (OR 1.07, CI95% [1.03–1.11], *p* < 0.001), F_S_O_2_ _*mean (day 1−3)*_ (OR 1.03 [1.00-1.06], *p* = 0.039), and right radial P_a_O_2_ _*mean (day 1−3)*_ (OR 1.03, CI95% [1.00-1.02], *p* = 0.023). On the contrary, ECMO duration and SAPS2 score were not independently associated with in-ICU mortality, and were therefore removed during the stepwise logistic regression analysis.

**Fig. 2:**
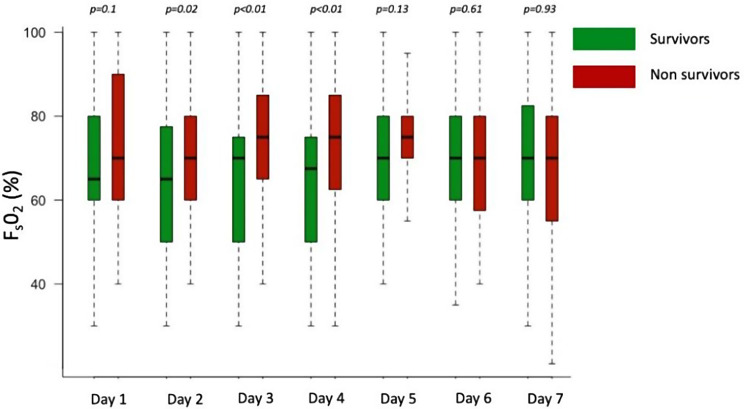
Day by day comparison of F_S_O_2_ according to outcome. F_S_O_2_ : Sweep gas oxygen fraction

Univariate and multivariate analysis of factor associated with in-ICU mortality is reported in Table 3. Day by day comparison of F_S_O_2_ according to outcome is presented in Fig. 2.

**Table 3:** Univariate and multivariate analyses of factors associated with in-ICU mortality among the 139 patients supported by peripheral VA ECMO

	Univariate analysis			Multivariate analysis	
Patients characteristics	Survivors (*n* = 87)	Non-survivors (*n* = 52)	p value	OR [CI 95%]	p value
Age (years)	52 [45;59]	62 [57;67]	**< 0.01**	1.07 [1.03–1.11]	**< 0.001**
SAPS2 score	56 [45;67]	72 [48;81]	**< 0.01**	**-**	**-**
SOFA score	10 [7;12]	11 [8;13]	0.23	-	-
*Center case-volume (VA ECMO/year)*					
< 30	20/87	12/52	0.28		
30–100	52/87	36/52		-	-
> 100	15/87	4/52			
ECMO duration (days)	6 [4;9]	7 [4;8]	0.6	-	-
F_S_O_2__* day 1*_ (%)	65 [60;80]	70 [60;90]	0.11	-	-
F_S_O_2__* day 2*_ (%)	65 [50;78]	70 [60;80]	**0.02**	**-**	**-**
F_S_O_2__* day 3*_ (%)	70 [51;75]	75 [65;84]	**< 0.01**	**-**	**-**
F_S_O_2__* mean (day 1−3)*_ (%)	67 [54;75]	72 [65;85]	**< 0.01**	1.03 [1.00-1.06]	**0.039**
F_S_O_2__* day 1 *_> 70%*	51/87	34/52	0.48	-	-
F_S_O_2__* day 2 *_> 70%*	49/87	37/49	**0.03**	**-**	**-**
F_S_O_2__* day 3 *_> 70%*	47/86	35/46	**0.02**	**-**	**-**
F_S_O_2__* day 1−3*_ > 70%*	39/87	33/52	**0.04**	**-**	**-**
Right radial P_a_O_2__* day 1*_ (mmHg)	120 [88;183]	165 [82;242]	0.21	-	-
Right radial P_a_O_2__* day 2*_ (mmHg)	99 [77;128]	114 [86;156]	0.06	-	-
Right radial P_a_O_2__* day 3*_ (mmHg)	94 [81;120]	109 [82;162]	0.08	-	-
Right radial P_a_O_2__* mean (day 1−3)*_ (mmHg)	111 [90;146]	141 [100;197]	**0.01**	1.03 [1.00-1.02]	**0.023**

Data are number and median [Interquartile range] OR: Odds ratio; CI95%: confidence interval; SAPS2: simplified acute; F_S_O_2_: Sweep gas oxygen fraction

* F_S_O_2 day X_ > 70% corresponds to at least one F_S_O_2_ value > 70% on day X

Bold values are p values < 0.05

## Discussion

In this multicentric cohort study of cardiogenic shock supported by VA ECMO, results may be summarized as follow: (1) median value of F_S_O_2_ _*mean (day 1−7)*_ was 70 [57; 79] %; (2) P_POST_O_2_ (a surrogate of lower body PO_2_) monitoring was infrequent and revealed significant hyperoxemia, and (3) higher F_S_O_2_ _*mean (day 1−3)*_ and right radial P_a_O_2_ _*mean (day 1−3)*_ were independently associated with in-ICU mortality.

Published data on F_S_O_2_ management are scarce. Most of studies report either only punctual F_S_O_2_ values [17, 18], or protocol for the initial F_S_O_2_ setting [1, 2, 5, 6, 19]. In a retrospective monocentric study on 54 patients, median F_S_O_2_ decreased from 80% [70–100] at baseline, to 70% [65–80] at 48 h [20]. In another study, mean F_S_O_2_ was around 80% between day 1 and day 10 of ECMO support [21]. In the study by Moussa et al., mean F_S_O_2_ ranged from 50% to 70% between baseline and day 2 [12]. Our data are in line with the previous studies, showing that F_S_O_2_ is usually set around 70%. Interestingly, we found that F_S_O_2_ varied inversely with ECMO center case volume. Because right radial P_a_O_2_ was also higher in low volume centers, we can hypothesize that low volume centers were more tolerant with hyperoxemia, leading to less down titration of F_S_O_2_ compared to most experienced centers. However, such observation needs to be interpreted cautiously because only 23% and 14% of patients were enrolled in low and high-volume centers. Then, this difference may reflect practices of a very few centers rather than a real case volume effect.

Using the threshold of 300 mmHg for the right radial P_a_O_2_, we found a quite low prevalence of severe hyperoxemia (12%). Our results are concordant with those of Jentzer et al. who found a 19.8% prevalence of severe hyperoxemia 24 h after ECMO start for cardiogenic shock [8]. This observation should probably be linked to an early F_S_O_2_ titration, as only 17% of patients of our cohort experienced at least one day with a F_S_O_2_ set at 100%.

Although P_POST_O_2_ was a main objective of the study, we found that P_POST_O_2_ monitoring was infrequent. It is important to distinguish P_POST_O_2_ monitoring, i.e. PO_2_ monitoring of the blood reinjected in the abdominal aorta which can be assimilated to the hepato-splanchnic PO_2_ monitoring, and functional membrane assessment evaluated once daily by increasing F_S_O_2_ transiently at 100% to determine the gas transfer capacity of the membrane. Some clinicians may consider P_POST_O_2_ monitoring useless, as right radial P_a_O_2_ may be sufficient to detect ECMO-induced hyperoxemia, and differential hypoxemia. In the subgroup of patients with available data, we found significant hyperoxemia with a median P_POST_O_2_ value of 198 mmHg, and two third of values above the ELSO recommended target of 150 mmHg [14]. Previously, only one study on 45 patients has reported data regarding to P_POST_O_2_. They found that median P_POST_O_2_ decreased from 301 [215–386] mmHg at baseline, to 140 [78–220] mmHg at H48. In this study, only one third of P_POST_O_2_ values were below 150 mmHg [20]. A possible reason for this tolerance with hyperoxemia might be the fear of unrecognized hypoxemia of the lower part of the body. Indeed, devices for continuous monitoring of P_POST_O_2_ or postoxygenator oxygen saturation exist but are not widespread. Then down titration of F_S_O_2_ might theoretically result in unknown low P_POST_O_2_, and hepato-splanchnic hypoxia [15]. One could also argue that for now, the safe P_POST_O_2_ target is still unknown, as randomized trials are ongoing [22].

Although in the setting of ECPR, most of studies have reported an association between P_a_O_2_ and outcome [2, 4–7, 9–11, 23], results are not so clear for patients with cardiogenic shock supported by VA-ECMO. Based on the ELSO registry, Munshi et al. did not found any association between P_a_O_2_ 24 h after ECMO start and mortality in the subgroup of 775 patients with cardiogenic shock [7]. In a smaller cohort by Ross et al., mean P_a_O_2_ of the 72 first hours was also not associated with mortality [1]. However, more recently, Moussa et al. reported that early hyperoxemia was associated with mortality in a cohort of 430 patients. In this study, mean P_a_O_2_, absolute peak P_a_O_2_, and mean daily peak P_a_O_2_ during the 48 first hours were associated with 28-day mortality [12]. An analysis of 9959 patients in the ELSO registry found an association between hyperoxemia (P_a_O_2_ > 150 mmHg 24 h after ECMO start) and in-hospital mortality [8]. Our results are in line with these findings.

Beyond the already described link between P_a_O_2_ and outcome, we found that F_S_O_2_ _*mean (day 1−3)*_ was independently associated with in-ICU mortality. Such finding is of interest because it may help to distinguish if hyperoxemia is a culprit or only a covariate [22]. Indeed, such link between hyperoxemia and prognosis might either be mediated by the proper harm of ECMO-associated hyperoxemia or be biased by the severity of cardiac failure. In the setting of peripheral VA-ECMO, the differential hypoxemia phenomenon results in heterogeneous PO_2_ along the aorta, depending of the location of the mixing zone [24]. In the most severe cardiac failure, the mixing zone is in the aortic arch, close to the brachiocephalic trunk. Then, right radial P_a_O_2_ is mainly determined by F_S_O_2,_ and its value is closed to the P_POST_O_2_ value. In the absence of measurement of stroke volume or its surrogates (pulse pressure or end tidal CO_2_ [25]), we cannot rule out that patients with higher right radial P_a_O_2_ were those with the most severe cardiac failure, having per se a higher mortality [26]. The fact that a higher F_S_O_2_ _*mean (day 1−3)*_ was independently associated with a higher in-ICU mortality supports the hypothesis of the proper harm of ECMO-induced hyperoxemia, as F_S_O_2_ may not be impacted by cardiac failure severity. Indeed, higher F_S_O_2_ _*mean (day 1−3)*_ may have resulted in higher P_POST_O_2_, and potentially more reperfusion injury of the gut [27], liver, and kidneys. Interestingly, in the study of Moussa et al., the mean F_S_O_2_ was lower in survivors than in non-survivors [12]. In the study of Justus et al., the median F_S_O_2_ also tended to be lower in survivors than in non-survivors (72% versus 78%) [21].

Our study has strengths. First, the multicentric design allowed us to detect a signal of variability of oxygenation practices according to center’s case volume. Second, we collected data during seven days after ECMO implantation, which corresponds to almost the whole duration of VA-ECMO support. We think that it is more adapted to study the real impact of ECMO-induced hyperoxemia than focusing on the first 24 h of support.

Our study has also several limitations. First, while we hypothesized that the association between F_S_O_2_ _*mean (day 1−3)*_ and in-ICU mortality was mediated by the P_POST_O_2_ value, we were unable to confirm it because P_POST_O_2_ monitoring was infrequent, even in high-volume centers. We also did not collect data on ECMO blood flow, which has been recently demonstrated to be a major determinant of right radial P_a_O_2_ [20]. Because the primary objective of the study was description of oxygenation practices, we also did not collect data allowing to evaluate the impact of ECMO-induced hyperoxemia on organ dysfunction. Second, our study was mostly conducted in France. Epidemiological data from another country may have led to different observation. Third, we did not find any association between centers case volume and in-ICU mortality. It is however commonly admitted that center’s case volume is associated with improved outcome [28]. These results might be explained by the limited sample size, the fact that most of patients were admitted in medium volume centers, and the short-term outcome (in-ICU mortality). Finally, due to the inclusion criteria (last 10 patients with cardiogenic shock supported VA ECMO for more than 24 h), data may reflect oxygenation practices over up to two years in low volume centers, compared to only 3 months in high volume centers.

## Conclusion

In a multicentric cohort study of cardiogenic shock supported by VA ECMO, median value of F_S_O_2_ _*mean (day 1−7)*_ was 70 [57; 79] %. P_POST_O_2_ monitoring was infrequent but revealed significant hyperoxemia. Higher F_S_O_2_ _*mean (day 1−3)*_ and right radial P_a_O_2_ _*mean (day 1−3)*_ were independently associated with in-ICU mortality. Based on these results, we can hypothesize that a strategy of systematic daily monitoring of P_POST_O_2_ may help to down titrate F_S_O_2_ and reduce ECMO-associated hyperoxemia and its potential deleterious effects.

### Electronic supplementary material

Below is the link to the electronic supplementary material.

Supplementary Material 1

## Data Availability

The datasets used during the current study are available from the corresponding author on reasonable request.
