# Supplementary material for: Oxygenation management during veno-arterial ECMO support for cardiogenic shock: a multicentric retrospective cohort study
Source: Ann Intensive Care. 2024 Apr 10;14:56. doi: 10.1186/s13613-024-01286-2 (PMC11006645; doi:10.1186/s13613-024-01286-2)
Supplement: Supplementary file 1 — Supplementary Material 1 [file 13613_2024_1286_MOESM1_ESM.docx]

Supplementary Table 1: Univariate and multivariate analysis of factors associated with ~~mean~~ F_S_O_2_ _mean (day 1-7)_ ≥ 70% among the 139 patients supported by peripheral VA ECMO.

Legend: Data are number and median [interquartile range] ~~mean (standard deviation)~~

Supplementary Figure 1: Daily evolution of mean P_POST_O_2_

Legend: Each color corresponds to one of the four centers measuring P_POST_O_2_ at its actual F_S_O_2_ value.

Supplementary Figure 2: *Daily evolution of oxygenation parameters according to center case volume and outcome*

Legend: a) corresponds to mean F_s_O_2_ between day 1 and 7 according to centers’ case volume; b) corresponds to mean F_s_O_2_ according to outcome; c) corresponds to mean right radial P_a_O_2_ between day 1 and 7 according to centers’ case volume; d) corresponds to mean right radial P_a_O_2_ according to outcome.

Supplementary Table 1: Univariate and multivariate analysis of factors associated with ~~mean~~ F_S_O_2_ _mean (day 1-7)_ ≥ 70% among the 139 patients under peripheral VA ECMO.

|  | Univariate analysis | | | Multivariate analysis | |
| --- | --- | --- | --- | --- | --- |
|  | F_s_O_2_ *_mean (day 1-7)_* < 70%  (n=67) | F_s_O_2_ *_mean (day 1-7)_* ≥ 70%  (n=72) | *p* value | OR [CI 95%] | *p* value |
| SAPS2 score | 61 [48;75] | 59 [46;77] | 0.6 | - | - |
| SOFA score | 10 [7;12] | 10 [7;13] | 1 | - | - |
| *Indication for VA ECMO* |  |  | 0.6 | - | - |
| Acute coronary syndrome | 25/67 | 25/72 |  |  |  |
| Cardiomyopathy | 24/67 | 20/72 |  |  |  |
| Postcardiotomy | 10/67 | 16/72 |  |  |  |
| Pulmonary embolism | 3/67 | 4/72 |  |  |  |
| Drug poisoning | 4/67 | 3/72 |  |  |  |
| Others | 1/67 | 4/72 |  |  |  |
| *Center case-volume (VA ECMO/year)* |  |  | **0.01** |  |  |
| > 100 | 13 /67 | 6 /72 |  | 1 | 1 |
| 30-100 | 46 /67 | 42 /72 |  | 1.34 [0.44 -4.47] | 0.6 |
| < 30 | 8/67 | 24 /72 |  | 5.04 [1.39 -20.4] | **0.017** |
| P_POST_O_2_ measurement | 23/67 | 16/72 | 0.19 | - | - |
| *Mechanical ventilation* |  |  |  |  |  |
| F_I_O_2_ *_mean (day 1-7)_* (%) | 42 [33;56] | 46 [36;64] | 0.08 | 1.01 [0.99-1.04] | 0.4 |
| Right radial P_a_O_2_ *_mean (day 1-7)_* (mmHg) | 105 [88;123] | 125 [97;163] | **<0.01** | 1.01 [1.00-1.02] | **0.006** |
| PEEP (cmH_2_O) | 7 [6;8] | 7 [6;10] | 0.1 | - | - |
| Extubation during ECMO | 28/67 | 19/72 | 0.07 | - | - |

Data are number and median [Interquartile range] OR: Odds ratio; CI 95%: confidence interval. SAPS2: Simplified acute physiology score; SOFA: Sequential organ failure assessment; F_I_O_2_: Inspired oxygen fraction; P_a_O_2_: arterial partial pressure of oxygen; PEEP: positive end expiratory pressure.

Supplementary Figure 1: Daily evolution of mean P_POST_O_2_

Each color corresponds to one of the four centers measuring P_POST_O_2_ at its actual F_S_O_2_ value.

Supplementary Figure 2: Daily evolution of oxygenation parameters according to center case volume and outcome


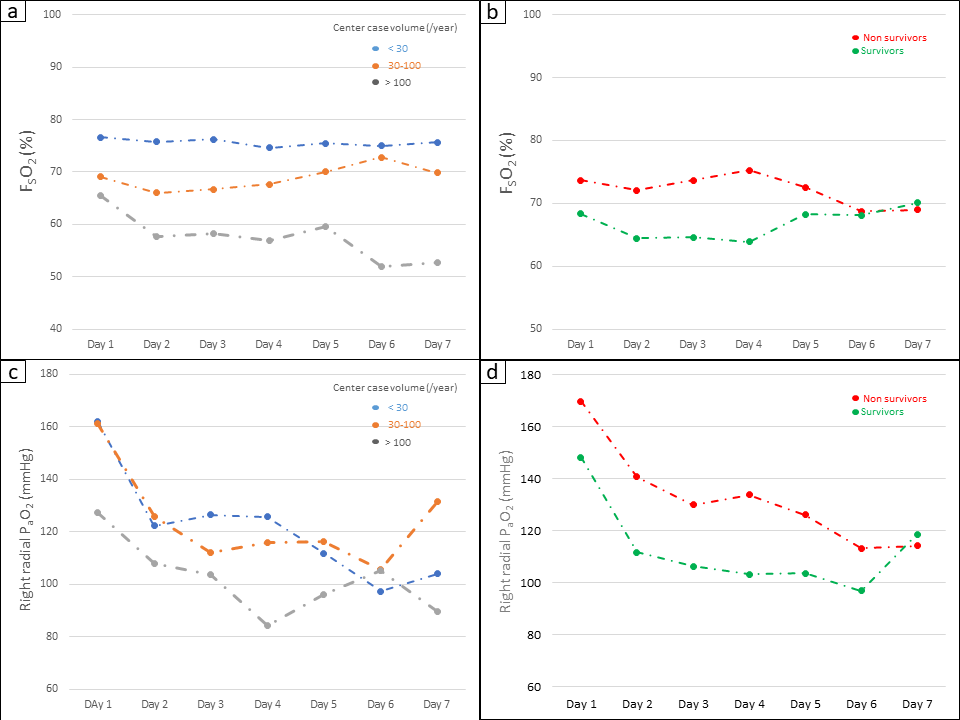


a) corresponds to mean F_s_O_2_ between day 1 and 7 according to centers’ case volume; b) corresponds to mean F_s_O_2_ according to outcome; c) corresponds to mean right radial P_a_O_2_ between day 1 and 7 according to centers’ case volume; d) corresponds to mean right radial P_a_O_2_ according to outcome.
